# In Search of Beautiful Molecules: A Perspective on Generative Modeling for Drug Design

**DOI:** 10.1021/acs.jcim.5c01203

**Published:** 2025-09-02

**Authors:** Remco L. van den Broek, Shivam Patel, Gerard J. P. van Westen, Willem Jespers, Woody Sherman

**Affiliations:** † Division of Medicinal Chemistry, Leiden Academic Centre for Drug Research, 225118 Leiden University, Einsteinweg 55, Leiden 2333CC, the Netherlands; ‡ 705916 Psivant Therapeutics, 451 D Street, Boston, Massachusetts 02210, United States; § Department of Medicinal Chemistry, Photopharmacology and Imaging, Groningen Research Institute of Pharmacy, Antonius Deusinglaan 1, Groningen 9713 AV, the Netherlands

## Abstract

Generative modeling with artificial intelligence (GenAI) offers an emerging approach to discover novel, efficacious, and safe drugs by enabling the systematic exploration of chemical space and to design molecules that are synthesizable while also having desirable drug properties. However, despite rapid progress in other industries, GenAI has yet to demonstrate clear and consistent value in prospective drug discovery applications. In this Perspective, we argue that the ultimate goal of generative chemistry is not just to generate “new” or “interesting” molecules, but to generate “beautiful” molecules—those that are therapeutically aligned with the program objectives and bring value beyond traditional approaches. We focus on five essential considerations for the successful applications of GenAI for drug discovery (GADD): 1) chemical synthesizability (accounting for time/cost constraints); 2) favorable ADMET (absorption, distribution, metabolism, excretion, and toxicity) properties; 3) desirable target-specific binding to modulate the biological mechanism of interest; 4) the construction of appropriate multiparameter optimization (MPO) functions to drive the GenAI toward the project objectives; and 5) human feedback from experienced drug hunters. Interestingly, defining the beauty of a molecule in a drug discovery program is not always obvious, being context-dependent as data emerge and priorities shift, making the role of expert human input indispensable. While MPO frameworks using complex desirability functions or Pareto optimization can help operationalize multifaceted project objectives, they cannot yet fully capture the nuanced judgment of experienced drug hunters. Reinforcement learning with human feedback (RLHF) offers a path to guide the GenAI toward therapeutically aligned molecules, just as RLHF played a pivotal role in training large language models (LLMs) like ChatGPT, especially in aligning the model’s behavior with human expectations. While not responsible for the model’s base knowledge, RLHF is essential in shaping how the model responds. In addition to RLHF, future progress in GADD will depend on better property prediction models and explainable systems that provide insights to expert drug hunters. “Beauty is in the eyes of the beholder”—for drug discovery, beauty is judged by experienced drug hunters and clinical success.

## Graphical abstract

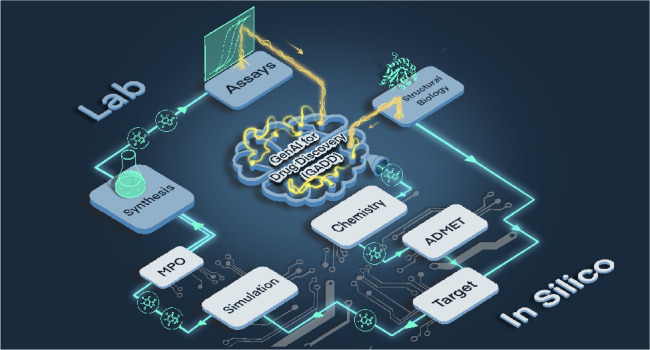

## Introduction

Generative AI (GenAI) has become the most rapidly adopted technology to emerge from the artificial intelligence (AI) revolution, making a significant impact across a broad range of industries,^1^ including content creation, marketing, customer support, software development, architectural design, manufacture prototyping, operations, and supply chain. However, GenAI for drug discovery (GADD) has been lagging. In this Perspective, we discuss the primary considerations for GenAI tools to meaningfully improve success rates in drug discovery. We hope that this Perspective is informative both for experienced drug hunters without AI expertise and AI engineers without drug discovery expertise. We focus on the current state of GADD, including what is working and why most models fail to produce what an experienced drug hunter might call “beautiful” molecules – those with therapeutic potential, synthetic practicality, and intuitive appeal based on a track record of bringing drugs to patients. First, we provide the context of GenAI in other fields, then dive into the drug discovery specifics.

The AI revolution has not happened overnight, as evidenced by decades of breakthrough advancements in fields such as image recognition,^2^ self-driving machines,^3^ computer vision,^4^ and self-learning algorithms.^5^ Now, GenAI is seeing a breakthrough in high-value use cases with both text and image generation models having become widely accessible to the public since the releases of DALL·E in 2021 and ChatGPT in 2022. This rise in GenAI for the casual consumer presented a unique change as compared with previous AI tools that have been geared toward experts. For the first time, the usage of AI has become mainstream. Simultaneously, businesses have rapidly adapted to accommodate and utilize these developments. For example, according to the McKinsey Global Survey on the State of AI in early 2024, merely two years after the widespread release of ChatGPT, 65% of businesses have adopted generative modeling in their operations.^6^ The fields of marketing and sales have seen the highest adoption of generative modeling, due to their direct benefit through applying the ready-to-use text and image generators. This is followed by product and service development, where the true potential of generative modeling comes to fruition as it drives innovation with the development of specialized generative models.^7^ Most industries have begun to invest significant time and resources into developing specialized generative models to address domain-specific challenges in their field.

In the field of drug discovery, GenAI has the potential to significantly accelerate the design of high-quality molecules tailored to specific targets and property objectives. However, despite growing interest and technological progress, the field remains in its early stages with limited validation that it meaningfully improves drug design, as measured by clinical success.^8^ Realizing the full value of GenAI in drug discovery will require overcoming substantial challenges, including ensuring that generated molecules are synthetically feasible, pharmacologically relevant, and have a positive impact on real-world outcomes (i.e., patients). Here, we emphasize the need for GenAI to produce molecules that are synthetically feasible and therapeutically aligned with the needs of a drug discovery program, which often evolves with the emergence of unexpected data and unanticipated changes in the competitive landscape. In generative chemistry, beauty reflects more than synthetic feasibility or numerical score – it captures the holistic integration of synthetic practicality, molecular function, and disease-modifying capabilities.

The qualitative notion of molecular beauty has long intrigued chemists. The Nobel Laureate in chemistry Roald Hoffmann opined on this topic over 3 decades ago in his paper “Molecular Beauty”,^9^ where he writes: “What makes molecules beautiful? It may be their simplicity, a symmetrical structure. Or it may be their complexity, the richness of structural detail that is required for specific function. Sometimes the beauty of a molecule may be hidden, to be revealed only when its position in a sequence of transformations is made clear. Novelty, surprise, utility also play a role in molecular aesthetics...”.

Before we discuss the various aspects of GenAI for drug discovery, we should first ask if there is a need for GenAI. One could argue that virtual high-throughput screening of ultralarge chemical libraries mitigates the need for GenAI.^10^ Indeed, screening large libraries of previously synthesized (or easy to synthesize) molecules has advantages, such as speed and costs, in contrast to the design and synthesis of *de novo* molecules, yet two significant limitations remain. First, the size of the ultralarge libraries is ever increasing, currently reaching up to 10^10^-10^11^ molecules in popular databases of virtual compounds such as GDB-17,^11^ and Enamine REAL Space^12^ which continue to expand the number of chemically diverse molecules.^10,13,14^ Screening these libraries becomes ever more computationally expensive/intractable, resulting in the need for focused library design strategies. Second, despite the seemingly large size of these libraries, they are relatively small and sparsely populated relative to the full drug-like chemical space, which is estimated at 1033 ^15^ to 10^60^.^16^ To partially mitigate these limitations, practices have been developed to *a priori* remove similar molecules, however, the explored chemical space remains fixed by the initial size of the virtual library.^17,18^ Furthermore, one of these removed “similar molecules” might offer a key breakthrough in the project, given that every atom matters in drug discovery and structure–activity relationships (SAR) are often rugged and nonlinear (e.g., activity cliffs). Navigating such complexity is where human chemists often recognize beauty, which is sometimes subtle, where serendipitous patterns in SAR point toward transformative chemical matter.

Significant progress has been made in the development of GenAI architectures that explore vast chemical spaces of synthesizable molecules. Architectures such as Variational Autoencoders (VAEs) enable smooth latent space navigation,^19,20^ Generative Adversarial Networks (GANs) facilitate realistic molecular generation,^21,22^ Reinforcement Learning (RL)-based methods allow for goal-directed optimization,^23–25^ and Transformer models capture intricate molecular relationships.^26,27^ These approaches are increasingly integrated into discovery workflows, with the aim of accelerating *de novo* molecule design through multiobjective optimization. However, challenges remain in addressing the gap between computational predictions and experimental measurements—the accuracy of most property prediction models is inadequate when exploring novel chemical spaces.

Beauty is in the eye of the beholder, and in drug discovery, every project carries its own unique goals, constraints, and trade-offs that shape the perception of beauty in a molecule. In this Perspective, we explore the three main pillars of successful molecular design: chemical synthesizability, ADMET (absorption, distribution, metabolism, excretion, and toxicity) properties, and target-specific bioactivity. We discuss strategies to balance and align these objectives through multiparameter optimization (MPO) and reinforcement learning with human feedback (RLHF). Given the deficiencies in existing predictive models and the vast nature of chemical space, we argue that human feedback is essential at this point for creating beautiful molecules. GenAI models can produce diverse synthesizable molecules, but we need more accurate models to predict ADMET, affinity, selectivity, and other drug-like properties if we want to remove humans from the loop in the design process.

We first address chemical space exploration, emphasizing the need for generative models to prioritize the practical aspects of procuring molecules, whether through vendor matching or synthesis. While vendor mapping can provide easy access to existing molecules, it inherently limits the scope of chemical space exploration. Therefore, the ability to generate synthetically practical molecules is crucial for ensuring that GenAI delivers experimentally testable molecules, a limitation that remains a bottleneck in the field. In addition, we highlight the potential for the integration of generative models with automated synthesis platforms, paving the way for closed-loop drug discovery. In these platforms, AI-generated molecules can be rapidly synthesized, tested, and refined in iterative cycles to accelerate optimization and improve success rates by generating project-specific data to improve the accuracy of predictive models. While automated chemistry broadly speaking is still in its infancy, subsets of reactions can be automated sufficiently such that closed-loop drug discovery can be tested (e.g., peptide chemistry).

Beyond procurement, we discuss the central role of property prediction models used to drive molecular generation. The accurate prediction of ADMET properties remains a challenge, making the early identification of liabilities through such models ineffective, often resulting in molecules with undesirable characteristics when tested in the lab. Furthermore, the incorporation of binding affinity (and off-target selectivity) into GenAI models has the promise to prioritize potent molecules, however accurate affinity and selectivity models tend to be too computationally expensive to run within a GenAI framework. As such, most applications of GenAI use crude scoring functions such as docking, which have known deficiencies that can be hacked by the GenAI objective function, as we will show in this work. Other target-specific properties such as kinetics and allosteric modulation are even more challenging and computationally intensive to predict. We explore how deep learning-based structure prediction, molecular docking, free energy perturbation (FEP) calculations, and molecular dynamics (MD) simulations can be integrated into generative frameworks to extend generative models beyond binding affinity alone. While the accuracy of these predictive models is central to the successful deployment, in many cases, the state-of-the-art predictive models alone are insufficient to steer generative algorithms toward “beautiful” molecules (Figure 1). These models often miss the nuanced interplay between structure, function, and clinical translatability that defines true molecular beauty in drug discovery.

**Figure 1:**
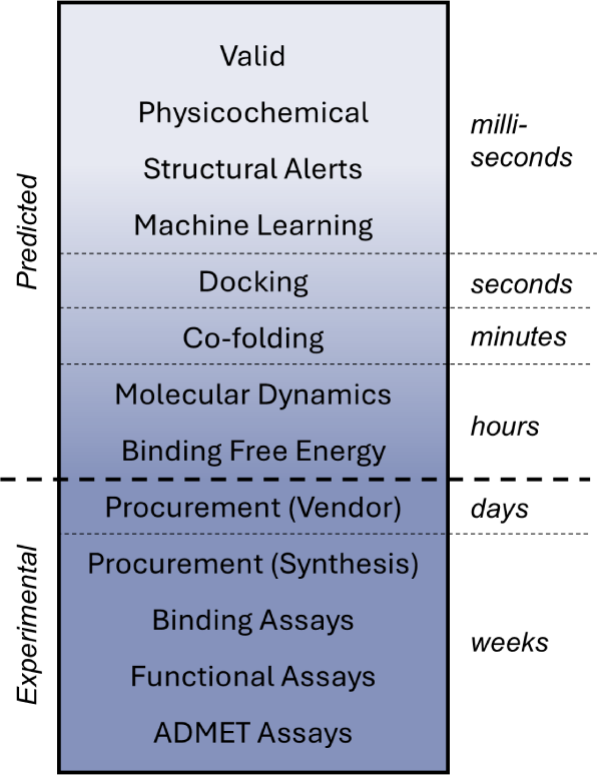
Oracles for assessing drug-like properties. The estimation of a molecule’s properties relevant to drug development is represented along a gradient. Experimental measurements remain the gold standard, but predictive oracles can serve as useful shortcuts. While longer runtimes often correlate with better performance, this relationship can vary significantly across methods and projects.

Given the complexity of balancing multiple, often competing, objectives, we emphasize the critical role of multiparameter optimization (MPO) in guiding the generative process. Constructing objective functions that balance multiple properties, such as synthetic feasibility, ADMET properties, affinity, and selectivity, remains a significant challenge. Techniques such as reinforcement learning (RL) and Pareto optimization approaches offer promising approaches for navigating these trade-offs. However, MPO models can sometimes be “cheated” generating molecules that formally conform to the objectives without truly aligning to the therapeutic goals of the project. This is especially problematic in cases where the predicted properties are inaccurate surrogates for real-world data from the lab. This highlights the continued importance of human oversight to ensure optimization efforts remain grounded in real-world drug discovery priorities. Through reinforcement learning with human feedback (RLHF), expert drug hunters provide essential guidance in refining objective functions, assessing molecular novelty, interpreting out-of-distribution predictions, and incorporating knowledge beyond what computational models can capture. Ultimately, it is this human expertise that guides GenAI models to align with the subjective definition of beauty in drug discovery. Just as GenAI for text or image creation depends on human feedback to define what is aesthetically pleasing or commercially valuable, generative chemistry requires an expert human-in-the-loop to define what is chemically, pharmacologically, and practically useful within the complex milieu of complex biology, constrained resources, competitive pressures, and the *je ne sais quoi* of designing a drug. In the end, the search for new medicines is not just a computational problem, but an aesthetic one in an ongoing pursuit of truly beautiful molecules that can make a positive impact on human health.

By integrating chemical feasibility, ADMET profiling, target-specific considerations, and expert-driven feedback into GenAI frameworks, the field can move toward a paradigm where computationally designed molecules have a higher likelihood of successful synthesis, biological relevance, and clinical translation. We conclude with a discussion on emerging trends, challenges, and future directions, emphasizing the need for more representative molecular embeddings, high-quality data sets, improved generative architectures, and robust validation strategies to fully realize the potential of GenAI for drug discovery (GADD).

## Synthesis and Procurement

### Introduction to Synthesis and Procurement

While the definition of “beautiful molecules” may be subjective, the generation of valid and synthetically accessible molecules is more measurable. Early efforts in GADD prioritized chemical validity through sequential representations such as simplified molecular-input line-entry specification (SMILES) and DeepSMILES, which provide a structured means of encoding chemical structures, but are prone to syntax errors that lead to chemically implausible molecules.^8^ To address these limitations, self-referencing embedded strings (SELFIES) was introduced to guarantee enforcing chemical validity at the representation level by design.^28^ However, despite these theoretical safeguards, SELFIES has not demonstrated significant practical improvements, likely due to its increased complexity, which can constrain model flexibility and limit molecular diversity.

Beyond sequential encoding, graph-based molecular representations directly model atomic connectivity but remain susceptible to generating chemically invalid structures, such as disconnected fragments or valency violations. Three-dimensional representations introduce additional complexity, often producing unrealistic conformations with steric clashes.^29^ Across two- and three-dimensional approaches, rigorous validation steps are required to ensure that generated molecules meet the standards of chemical feasibility. Furthermore, stereochemistry, although frequently overlooked, must be explicitly addressed, as it greatly influences biological activity, receptor binding, and metabolic stability. Incorporating stereochemical constraints improves the relevance of generated molecules and enhances their potential downstream applicability in drug discovery. While the generation of chemically valid molecules suffices for *in silico* applications such as machine learning and virtual screening, advancing molecules toward experimental validation introduces new challenges. Validity alone does not guarantee synthetic feasibility or commercial availability. To move from computational predictions to real-world testing, molecules must be procurable within defined time and cost constraints.

In this section, we examine current, emerging, and future approaches to molecular procurement and synthesis, emphasizing their roles in overcoming practical barriers to experimental validation and in turn improving the predictive power of generative models. We will explore procurement strategies ranging from chemical vendors to automated design platforms, highlighting how their integration with generative modeling can accelerate the discovery of novel drugs by expanding accessible chemical space and validating computational predictions through experimental evaluation.

### Vendor Mapping

The procurement of molecules remains a significant constraint in the drug discovery process. In traditional medicinal chemistry, molecules are often designed around a known scaffold, with modifications introduced at specific side positions. However, this paradigm does not necessarily apply to molecules generated via machine learning (ML) methods, which can exhibit high structural diversity without a singular, easily reducible scaffold. This increased diversity poses challenges for direct procurement and necessitates efficient strategies for identifying synthesizable analogs.

Figure 2A shows the main approaches available for the procurement of generated molecules. Sometimes one can directly purchase the molecules of interest from a vendor. However, this is often not the case, given the vastness of chemical space and the limited number of previously synthesized molecules. One widely used approach to address this challenge is vendor mapping, which involves searching chemical vendor inventories for molecules closely resembling computationally generated structures. When the primary goal is to match a predefined distribution of molecular properties, similarity-based methods such as nearest-neighbor searches using Tanimoto similarity can be employed. These approaches enable rapid identification of commercially available molecules that approximate the desired molecular characteristics. For molecules optimized using structure-informed techniques, additional similarity measures are required to avoid losing relevant structural properties. Pharmacophore and/or shape matching approaches offer an alternative strategy, allowing researchers to identify the closest structurally similar molecules within vendor databases based on three-dimensional alignment rather than purely molecular fingerprints.

**Figure 2:**
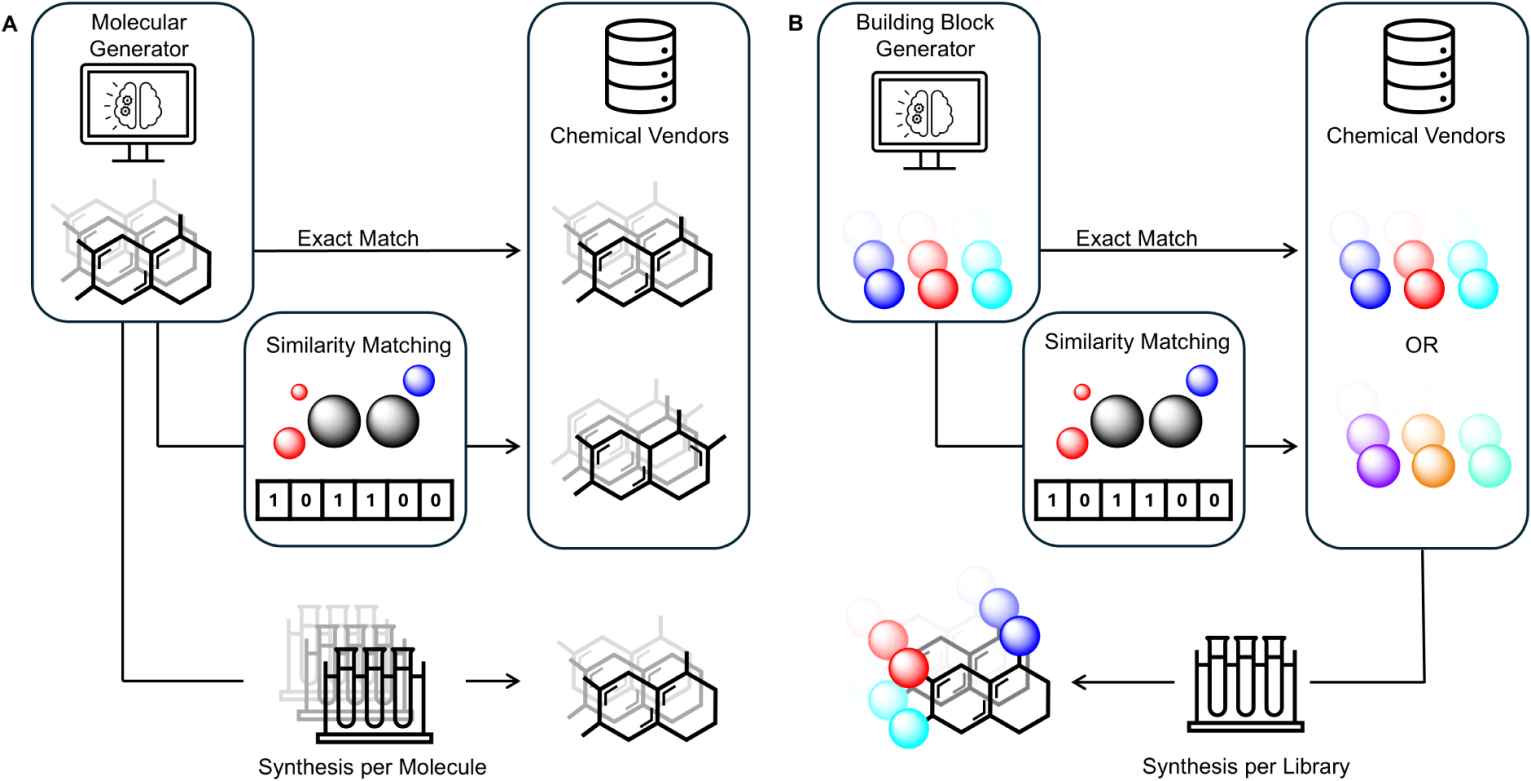
Approaches to chemical procurement of generated molecules. (A) Generated molecules can be obtained through bespoke synthesis of each molecule. Alternatively, generated molecules can be matched to vendor libraries where identical or similar molecules are purchased, although substantially similar molecules may not be available. (B) Generated building blocks are matched to the closest available option in the vendor library, followed by combinatorial synthesis to ensure large-scale synthesis without requiring extensive synthesis per molecule.

Despite its ease of use, vendor mapping has inherent limitations, primarily due to the finite size of chemical vendor inventories. While the number of commercially available molecules continues to grow, with Enamine, for example, surpassing 10^9^ molecules,^12^ this still represents only a minute fraction of the total theoretically synthesizable chemical space. Consequently, while vendor mapping is an efficient means of procuring molecules from well-characterized chemical space, it is often insufficient for obtaining molecules that are identical or close enough to the computationally generated molecules. In cases where precise structural replication is preferred or necessary, alternative synthesis-driven strategies must be explored.

### Combinatorial Libraries

The high diversity of molecules coming from GenAI often present challenges for synthesis. Combinatorial libraries offer a practical solution by enabling the systematic generation of large molecular sets through predefined synthetic strategies. These libraries consist of well-characterized building blocks combined using standardized, often simple reactions, allowing for the rapid synthesis of up to 10^6^ molecules while covering specific regions of chemical space.^30^ Additionally, when integrated with vendor mapping for procurement of building blocks, combinatorial library approaches provide an efficient means of procuring diverse molecular collections in a cost-effective and scalable manner (see Figure 2B).

The selection of building blocks for combinatorial libraries is subject to specific constraints, primarily dictated by the reaction chemistry employed. While this approach increases efficiency and scalability of synthesis, it limits the accessible chemical space. Functional group compatibility, stability under reaction conditions, and molecular size must all be carefully considered to ensure successful synthesis. Moreover, additional constraints may be introduced based on the research objective. To maximize chemical diversity, GenAI can be employed to design building blocks that span a broad range of chemical space, although deviating from the commercially available building blocks results in novel synthesis (hence the efficiency/scalability issues noted above). Conversely, if a more targeted chemical space is desired, such as when optimizing for a known binding pocket, building block selection can be biased toward structures that demonstrate predicted binding enrichment. However, for combinatorial library design with commercial building blocks, GenAI generally does not offer an advantage over traditional enumeration pipelines until the library space reaches a scale where exhaustive enumeration becomes computationally infeasible.

At the same time, automated closed-loop synthesis platforms are particularly well-suited for executing combinatorial library strategies.^31^ Automated design-make-test-analyze (DMTA) workflows enable iterative and efficient exploration of constrained regions of chemical space, which might be suitable for a given problem of interest. Bayesian optimization techniques can be applied to refine reaction conditions in these systems, particularly when the underlying chemistry remains similar across library members. This simultaneously accelerates reaction optimization and ensures reliable, reproducible synthesis at scale. Since combinatorial libraries typically rely on a limited number of well-characterized reactions, automated platforms can rapidly execute large-scale library production while integrating analytical feedback in real time, thereby closing the loop between molecular design and experimental validation.

While automation has not broadly generalized to cover the vast chemical space of interesting medicinal chemistry, there are certain chemistries that have already been put to practice, such as peptide chemistry. Given the well-defined nature of the building blocks (amino acids) and the relevant chemistry (amide coupling), peptides are well suited for closed-loop automated DMTA cycles. Peptide chemistry offers a compelling solution to the retrosynthesis challenge by providing a highly modular and predictable framework for synthesis and can often proceed with minimal optimization, making it ideally suited for scenarios requiring rapid, high-throughput generation of candidate molecules. Solid-phase peptide synthesis (SPPS) enables automation and parallelization, streamlining the production of diverse peptide libraries for screening.^32^ However, peptides come with inherent therapeutic limitations, such as poor cell permeability, rapid degradation by proteases, and limited oral bioavailability,

### Retrosynthesis

Traditionally, GenAI has been viewed as part of a process that works in close conjunction with retrosynthesis. The ideal scenario of GADD would involve generating a desirable molecule and directly synthesizing it, thereby providing the optimal molecule as the solution to the problem at hand. However, this approach introduces a significant reliance on advancements in the field of computer-aided synthesis prediction (CASP), which remains a developing area of research. Current tools have made notable progress in deconstructing molecules into their constituent building blocks, providing valuable insights to medicinal chemists about where to begin their synthesis efforts. While these tools can suggest likely reactions and starting points for molecular construction, they do not fully equip researchers (or robots) to directly synthesize generated molecules.^33^ The information provided is still limited and often requires a high degree of expertise and manual intervention from chemists to complete the synthesis. Crucial factors such as the specific reactants and reaction conditions required for successful synthesis remain largely overlooked within current approaches.

Significant strides are being made in closed-loop synthesis, where reaction conditions are predicted using advanced techniques like Bayesian optimization, yet challenges persist in accurately predicting which specific conditions should be employed for a given molecule synthesis. Despite the progress in predicting reaction pathways, the ability to forecast the optimal parameters for any given reaction remains a considerable hurdle. This gap continues to hinder the seamless integration of generative modeling with retrosynthesis in the context of drug discovery, underscoring the need for further advancements to fully realize the potential of GenAI in practical applications where one wishes to quickly synthesize the most desirable/beautiful molecules.

### Exploring Other Chemical Spaces

Natural product synthesis opens up a vast and chemically diverse space, tapping into the full spectrum of what is theoretically achievable.^34^ Natural products, mostly produced through biosynthetic gene clusters (BGCs), offer a rich source of novel molecules with unique biological activities. Approaches like metabolic engineering and biocatalysis can be used to harness these pathways, enabling the efficient synthesis of complex molecules that might be difficult to achieve through traditional organic chemistry. Both *in vitro* and *in vivo* synthesis methods allow for the production of these natural products, providing flexibility in how these molecules are generated. Additionally, expanding into areas such as unnatural amino acids and macrocycles broadens the chemical diversity even further. Natural products are known for their distinct pharmacological properties, making them valuable in drug discovery.

### Active Learning on Synthesized Molecules

Synthesizability is a critical prerequisite for generative molecular design, but it is far from sufficient. Therapeutically relevant molecules must also align with project-specific objective functions, including potency, selectivity, pharmacokinetics (PK), and toxicity. These multifactorial criteria define the fitness landscape for a given drug discovery campaign and serve as essential constraints in molecular design, guiding prioritization and optimization beyond chemical tractability alone. The optimal use of GenAI for drug discovery arises when active learning frameworks are applied continuously throughout a project. Active learning couples predictive modeling with experimental validation in a closed-loop system that prioritizes the synthesis and testing of molecules expected to yield maximal information gain and project progression. As new molecules are synthesized and experimentally characterized, the resulting data feed back into the model training process, refining both the generative and predictive components of the design loop.^35^ This iterative DMTA cycle facilitates more efficient exploration of chemical space, focusing synthetic efforts on regions that are not only synthetically accessible, but also enriched for molecules likely to meet therapeutic objectives. By embedding active learning into generative pipelines, drug discovery efforts can dynamically balance exploration and exploitation—accelerating lead optimization and improving the overall quality of candidate molecules. In the next sections, we will discuss the various approaches for ADMET and target-specific predictions, along with the strengths/limitations of MPO frameworks.

## ADMET Properties in Generative Models

### Introduction to ADMET

In drug discovery, the optimization of absorption, distribution, metabolism, excretion, and toxicity (ADMET) properties is critical for the successful translation of small molecules from early stage hits to clinical candidates. Poor ADMET characteristics are a leading cause of late-stage drug failures, underscoring the importance of integrating ADMET considerations into lead optimization. Absorption dictates the extent and rate at which a compound enters systemic circulation, with key factors including solubility, permeability, and transporter interactions such as efflux by P-glycoprotein (P-gp). Distribution determines how the drug is partitioned within the body, influenced by plasma protein binding, volume of distribution, and tissue penetration, particularly for molecules targeting the central nervous system, where blood-brain barrier permeability is a major constraint. Metabolism, primarily governed by hepatic cytochrome P450 (CYP450) enzymes, modulates drug clearance and bioavailability through oxidative and conjugative pathways, which can either enhance elimination or generate bioactive or toxic metabolites. Excretion, occurring via renal and hepatic routes, is dictated by metabolic stability, renal clearance mechanisms, and biliary elimination, collectively influencing systemic drug exposure and half-life. Toxicity remains a major challenge in drug development, with off-target interactions leading to hepatotoxicity, cardiotoxicity (e.g., hERG inhibition), or genotoxicity. Machine learning (ML) and physics-based predictive models have promise to accurately predict ADMET properties, although significant gaps exist in both the quantity and quality of training data. Publicly available data sets often suffer from noisy, inconsistent measurements, limited chemical diversity, and assay variability between laboratories. Even when using the same nominal protocol, a given assay can produce variable results across different experimental settings. These limitations significantly degrade model reliability, especially for novel compounds that lie far from the training distribution.

Early stage ADMET prediction can help reduce late-stage failures in drug development, where poor pharmacokinetics and safety profiles are among the most common reasons for attrition. Late-stage failures are particularly costly due to the significant investment required for preclinical and clinical studies; thus, integrating ADMET assessments during hit-to-lead and lead optimization phases can enhance the probability of clinical success. For example, a common metric to indicate the potential for nonspecific binding (often leading to undesirable pharmacological effects) is the ligand lipophilic efficiency (LLE).^36^ Computational approaches including machine learning models, quantitative structure–activity relationship (QSAR) methods, and physics-based simulations have been developed for predicting key ADMET properties such as solubility, permeability, metabolic stability, and toxicity. These predictive models can be used to help prioritize molecules with favorable pharmacokinetics and safety profiles, although model errors can lead to reward hacking in GenAI frameworks. Most ADMET models break down when exploring chemical spaces not closely related to the training data.

### ADMET in GADD

Unlike traditional medicinal chemistry approaches, which rely on iterative synthesis and screening, GenAI leverages deep learning (DL) architectures such as reinforcement learning (RL), variational autoencoders (VAEs), and generative adversarial networks (GANs) to explore vast chemical spaces efficiently. These models integrate multiparameter optimization (MPO) functions that can consider physicochemical constraints, bioavailability, metabolic stability, and toxicity risks alongside potency and selectivity. By incorporating predictive ADMET models, ranging from ML-based QSAR models to physics-informed simulations (see Figure 1), generative algorithms can penalize undesirable properties such as poor solubility, high plasma protein binding, or CYP450-mediated metabolic instability, thereby biasing molecular generation toward drug-like and progressible candidates. Furthermore, the iterative refinement of generative models through active learning and experimental feedback loops can enhance their predictive power, thereby increasing the probability that proposed molecules will align with real-world project requirements, i.e., “beautiful molecules”. This data-driven approach with predictive models has the potential to accelerate lead optimization, minimize synthetic dead ends, and reduce the risk of late-stage failures due to poor pharmacokinetics or toxicity, ultimately improving the efficiency of drug development pipelines. However, it is important to recognize that predictive power is tightly constrained by the training domain. In practice, many GenAI efforts generate novel molecular scaffolds and functional groups that are poorly represented – or completely absent – in public or proprietary ADMET data sets. As a result, the accuracy of model-guided generation is often overestimated, particularly in exploratory programs where generalizability matters most.

### Predictive Models for ADMET Properties

Computational approaches for ADMET have been widely used in modern drug discovery, although significant challenges still exist, especially when deviating from the domain of their training data.^37^ High-quality ADMET models can enable the early assessment of PK and safety properties to reduce late-stage failures. These methods range from data-driven ML models to physics-based simulations and hybrid approaches that combine empirical and mechanistic insights. ML-based models, including QSAR models, DL architectures, and graph neural networks (GNNs), leverage large-scale experimental data sets to predict key properties such as solubility, permeability, metabolic stability, and toxicity. Molecular docking and molecular dynamics (MD) simulations provide structural insights into drug-enzyme interactions, such as for metabolism by CYP450 enzymes and transport via efflux proteins like P-gp.^38^ Additionally, quantum mechanical (QM) calculations can be used to understand reactivity and bioactivation pathways relevant to drug metabolism.^39^ Hybrid approaches, such as ML-augmented physics-based models, can enhance prediction accuracy by incorporating experimental feedback and structural insights. The integration of predictive ADMET models within AI-driven generative chemistry frameworks enable the design of molecules with improved PK and toxicity profiles, accelerating lead optimization. As computational power and data set quality continue to improve, these *in silico* methodologies will become increasingly reliable, ultimately enabling more efficient and rational drug design. However, it is important to reiterate the challenges of predicting ADMET properties, especially for novel molecules in regions of chemical space that deviate from available training data. This is particularly problematic in GenAI workflows, where exploration is a feature, not a bug. Ironically, the very strength of generative models (their ability to innovate) renders them susceptible to proposing molecules that exploit blind spots in ADMET predictors, leading to compounds that “look good” to the model but fail in real-world assays.

#### ML-Based ADMET Models

Despite promising advances in model architectures, the ultimate bottleneck in ADMET prediction remains the data. No matter how sophisticated the algorithm, a model trained on flawed or narrow data will produce misleading predictions when applied to novel compounds. Traditional random forest (RF) models, which are ensemble-based decision tree algorithms, have been widely used due to their ability to handle high-dimensional chemical descriptors and provide interpretable feature importance rankings. More recently, DL architectures, including convolutional neural networks (CNNs) and recurrent neural networks (RNNs), have demonstrated superior performance in capturing nonlinear relationships between molecular structure and ADMET properties by learning hierarchical feature representations directly from molecular graphs or SMILES strings. GNNs, an emerging class of DL models, further enhance predictive capabilities by representing molecules as node-edge graphs, where atoms and bonds encode chemical and electronic properties. Unlike traditional descriptor-based methods, GNNs can model complex structural and topological interactions, making them particularly well-suited for predicting protein–ligand interactions, metabolic transformations, and toxicity mechanisms.

However, the performance of any ML model is fundamentally limited by the quality, diversity, and relevance of the data it is trained on. Many models suffer from data bias, limited chemical coverage, and poor generalizability, particularly when applied to new chemical series, novel targets, or complex polypharmacological settings. In these regimes, training data may be sparse, noisy, or even contradictory. As a result, expanding access to larger, more diverse, and high-quality ADMET data sets is essential for improving model robustness and practical utility in real-world drug discovery scenarios. Alternatively, hybrid ML frameworks that combine pretrained embeddings, transfer learning, and active learning strategies are being developed to overcome data sparsity issues in ADMET modeling. As ML models continue to evolve, their integration with generative chemistry and multiparameter optimization (MPO) frameworks is expected to drive the rational design of molecules with improved pharmacokinetic and safety profiles, accelerating drug discovery efforts.

#### Physics-Based ADMET Models

Structure-based approaches like molecular docking have been used to predict metabolism by modeling the binding of small molecules to metabolizing enzymes, such as CYP450, to estimate substrate specificity, metabolic stability, and potential bioactivation pathway.^38^ More recently, co-folding methods like AlphaFold,^40^ Boltz,^41^ and Chai^42^ have emerged as competitive with traditional docking. These docking/co-folding approaches leverage scoring functions that approximate binding affinity and interaction geometry, which can provide insights into enzymatic processes. Additionally, molecular dynamics (MD) simulations can be used to refine these predictions by capturing protein flexibility, dynamic ligand-enzyme interactions, and binding free energies, offering a more physiologically relevant view of metabolism. Beyond docking and MD, quantum mechanics (QM) methods can provide an atomistic understanding of molecular reactivity, particularly for assessing electronic properties, redox potential, and potential toxicophores. Density functional theory (DFT) calculations are commonly applied to predict reaction mechanisms relevant to phase I metabolism, such as oxidation, hydrolysis, and reduction, as well as phase II conjugation reactions. These physics-based models complement ML approaches by providing mechanistic insights that enhance the interpretability and generalizability of ADMET predictions. However, structure-based methods tend to be computationally intensive and require domain expertise in order to obtain useful results. Furthermore, even with domain expertise and sufficient computational resources, simulating protein–ligand interactions is a complex modeling exercise that has a high level of uncertainty. While insights and accurate predictions can be obtained, the user should be aware of limitation of these methods. As computational efficiency and accuracy continue to improve, we expect to see the integration of docking/co-folding, MD, and QM models with AI-driven drug design pipelines is expected to enhance the predictive power of ADMET assessments, leading to more robust early stage drug candidate selection. Currently, the more accurate structure-based approaches are too computationally intensive to be used within the inner loop GenAI reinforcement learning frameworks, but can be used as a final filter before purchasing or synthesizing compounds for testing.

#### Data Sets for Training and Validating Models

The quality of data sets used to train ADMET prediction models is a critical determinant of their reliability and domain applicability. Unfortunately, many publicly available data sets are riddled with inconsistencies, including heterogeneous assay formats, variable experimental conditions, and nonstandardized endpoints. Even when data are labeled similarly, interlaboratory variability can result in substantial differences in measured values, introducing label noise that reduces model confidence.^43^ For more complex endpoints, such as transporter-mediated permeability, hepatic clearance, or drug-induced liver injury, the lack of standardized, reproducible assays makes high-quality data even more scarce. These limitations reduce the effective training domain for models, leading to overfitting on narrow chemical scaffolds and brittleness in novel applications.

To overcome these limitations, *in silico*-generated data, including MD simulations, QM calculations, and structure-based docking/co-folding can be used to augment experimental data sets and fill data gaps. However, such computational predictions introduce their own uncertainties, as model-derived data may reinforce preexisting biases or propagate errors from upstream algorithms. Hybrid approaches, where machine learning (ML) models are trained on a combination of experimental and high-fidelity *in silico* data, have shown promise in improving model robustness.^44^ Active learning strategies that selectively prioritize the most informative experimental measurements for model retraining can further enhance predictive performance. Ultimately, ensuring high data set diversity, rigorous data curation, and integration of orthogonal experimental and computational sources is essential for developing both ML and physics-based ADMET models that are accurate and applicable across a broad chemical space in generative chemistry pipelines.

### Future Directions in ADMET Modeling for Generative Chemistry

*In silico* ADMET models are integral to generative chemistry workflows, enabling the rapid assessment of pharmacokinetic and safety properties early in the drug design process. Their primary strengths lie in speed, offering fast, scalable alternatives to traditional time-consuming experimental assays. ML-based models, including RF, DL, and GNNs, leverage large data sets to predict key properties such as solubility, permeability, metabolic stability, and toxicity with increasing accuracy. Complementary physics-based approaches, such as molecular docking/co-folding for metabolism and quantum chemistry for reactivity, offer mechanistic interpretability and improved generalizability across chemical space. The integration of these predictive models into multiparameter optimization (MPO) frameworks enables the concurrent evaluation of ADMET, potency, and synthetic tractability, ensuring that generated molecules are not only efficacious, but also developable. Together, these tools form the foundation for next-generation generative pipelines that can propose high-quality, balanced candidates with fewer design cycles.

Despite their utility, current *in silico* ADMET models face notable limitations. Many suffer from data bias and limited domain applicability, often stemming from imbalanced or sparse training data sets that fail to represent the full breadth of chemical space or only contain categorical values (e.g. <10 μM for hERG). Prediction accuracy is further constrained by the quality of input data that comes from experimental noise, inconsistent assay protocols, and undersampled concentration measurements, all of which can degrade model performance. More complex properties, such as idiosyncratic hepatotoxicity or drug-drug interactions, remain especially challenging due to their multifactorial and poorly understood nature. Worse yet, in low-data regimes or with poorly calibrated models, generative algorithms may exploit weaknesses in ADMET predictors (a phenomenon known as reward hacking). This can yield molecules that appear optimal to the model but fail to meet efficacy or safety requirements in the lab. Addressing this requires a combination of uncertainty-aware modeling, expert oversight, and continual feedback from real-world experimental data.

Looking ahead, next-generation ADMET modeling in generative chemistry will require adaptive, context-aware frameworks capable of evolving with program objectives. Integrating reinforcement learning with human feedback (RLHF) offers a promising path to align generative models not only with quantitative scoring functions but also with qualitative medicinal chemistry intuition. Advances in molecular representation learning, uncertainty quantification, and explainable AI (XAI) will further enhance model reliability and interpretability. Broadening the applicability of these models will depend on access to high-quality, federated data sets and the incorporation of real-time experimental feedback loops to improve generalization across chemical space. Ultimately, designing high-quality molecules—those that are synthetically practical and therapeutically aligned will hinge on the seamless integration of predictive ADMET modeling, interactive generative tools, and expert human oversight. This convergence of data-driven methods and domain expertise is essential to unlock the full potential of AI in drug discovery.

## Predicting Target-Specific Properties

### Introduction to Binding Affinity Predictions

Another critical pillar in rational drug design is the prediction of binding affinity and selectivity toward one or multiple protein targets. This property is essential to ensure that a molecule will effectively engage its intended target and avoid unintended interactions that can lead to side effects or toxicity. In polypharmacology, this task becomes even more complex, as molecules must be assessed across a panel of proteins to identify potent molecules with relevant proteins, while maintaining minimal off-target activity.

Historically, two major approaches have emerged for predicting binding affinity: ligand-based and structure-based methods. Ligand-based methods rely on known activity data and molecular descriptors to train ML models that can predict bioactivity without requiring protein structures. Among these ligand-based models, Quantitative Structure–Activity Relationship (QSAR) modeling uses statistical and ML methods to predict potency, selectivity, and toxicity, one target at a time. The ligand information can be represented by physicochemical descriptors (e.g., molecular weight, log P) or molecular fingerprints (e.g., ECFP, MACCS). Proteochemometric (PCM) models extend QSAR by incorporating protein information, either using multiple sequence alignment-based descriptors or embeddings, enabling predictions across protein families and even accounting for mutations.^45,46^ While ligand-based machine learning models offer near-instant predictions and are computationally inexpensive, their applicability is constrained by the chemical and biological space covered in their training data, limiting reliability when extrapolating to novel scaffolds or targets without sufficient prior examples.

Structure-based methods such as molecular docking, co-folding, and molecular dynamics simulations can simulate the physical interaction between a ligand and its target protein, estimating binding strength through shape complementarity, hydrogen bonding, and other molecular interactions.^47,48^ Docking enables high-throughput screening of large virtual libraries but scoring functions are typically rough approximations of binding free energy, and accuracy is highly sensitive to input structure quality and protein flexibility. To overcome the limitations of docking, particularly its reliance on static structures, physics-based methods such as molecular dynamics (MD) simulations offer a dynamic view of ligand-protein interactions by capturing molecular flexibility and behavior over time.^49^ Relative Binding Free Energy (RBFE) and Absolute Binding Free Energy (ABFE) methods (collectively called Free Energy Perturbation or FEP here) offer more accurate affinity estimates, outperforming docking in many prospective applications.^50,51^ However, these improvements come at a steep computational cost. Rigorous FEP simulations may require dozens of GPU hours per compound, rendering them infeasible for high-throughput screening or iterative reinforcement learning loops within GenAI frameworks for drug discovery. Moreover, their success still depends heavily on expert system setup, accurate protonation states, and high-resolution input structures—all of which present major barriers to automation.

### The Emergence of Co-folding

Co-folding techniques represent a new approach to predicting binding poses and binding affinity by modeling the ligand and protein as a joint system rather than treating one as fixed. Unlike traditional docking, co-folding captures the mutual structural adaptation between a ligand and its target, enabling predictions of binding modes where the protein conformation is ligand-dependent. However, co-folding models remain in their infancy and typically do not yet achieve the quantitative accuracy required for fine-grained affinity ranking. Additionally, their performance is highly dependent on the availability of high-quality training data, which is sparse for the targets of greatest interest (i.e., undrugged targets).^52^ While FEP (and MD more generally) can account for such dynamics with high accuracy, they typically require hours to days per molecule, making them impractical for large-scale or iterative workflows. In contrast, recent co-folding models can produce physically plausible protein–ligand complexes within minutes.^29,40,41^ Crucially, the rapid inference time of co-folding makes it a natural fit for *de novo* molecule generation pipelines. When integrated with generative models, co-folding enables real-time structural evaluation of candidates during the design process, allowing for direct feedback on binding plausibility and pose quality. This dynamic, structure-aware generation loop supports the creation of molecules tailored to flexible binding pockets or noncanonical targets, greatly expanding the chemical design space accessible to rational drug discovery.

### Beyond Binding Affinity: Kinetics and Functional Effects

While achieving a requisite level of binding affinity has been a longstanding objective in drug discovery, the target-based paradigm has since expanded to include binding kinetics, covalent engagement, and functional modulation—properties that better capture the complexity of therapeutic efficacy and target engagement *in vivo*. Indeed, biology does not exist in equilibrium and binding kinetics are increasingly recognized as key predictors of drug efficacy. By analyzing association (*k*_on_) and dissociation (*k*_off_) rates, researchers gain insights into residence time, an important factor influencing therapeutic duration and selectivity.^53^ While MD simulations offer a framework to study these kinetic processes by modeling transitions between bound and unbound states, accurately predicting rate constants from simulation remains computationally demanding and methodologically challenging. Covalent binding represents another mode of binding kinetics where the drug forms a stable bond with its target, effectively removing the need to consider dissociation rates. This irreversible or quasi-irreversible interaction can dramatically prolong residence time and enhance efficacy, particularly relevant in cancer and enzyme inhibition. However, covalent strategies require careful optimization to avoid off-target reactivity and ensure selective engagement.^54^

Further complexity arises when moving beyond binding kinetics and covalency to consider functional outcomes such as allostery, agonism, and downstream signaling. Allosteric modulators act at sites distinct from the active site, offering increased selectivity and modulation of activity. Agonists, inverse agonists, and partial agonists provide additional layers of control, enabling nuanced modulation of therapeutic responses that can optimize *in vivo* efficacy and minimize side effects.^55^

### Incorporating Phenotypic Endpoints

The increasing complexity of therapeutic mechanisms has driven the need for broader, more integrative approaches. Polypharmacology, where drugs interact with multiple proteins, can provide synergistic benefits in treating complex diseases like cancer or neurological disorders but poses challenges in managing specificity and side effects.^56^ An emerging paradigm shift in drug discovery calls for the integration of phenotypic observations with target-specific modulation. This mechanistic biology approach, as highlighted by Sadri,^57^ critiques reductionist models that rely solely on single modulation to produce the desired therapeutic effect. Instead, it promotes evidence-based strategies that incorporate system-level and phenotypic data to capture causal relationships between drug action and therapeutic effect. While such integration remains a challenge in current GenAI workflows, it represents a promising direction for future advancements.

### Limitations of Rigorous Structure-Based Methods in Generative AI

Despite its central importance, binding affinity is one of the hardest drug-like properties to predict with high accuracy. Most real-world targets exhibit conformational flexibility, water-mediated interactions, and other complex behaviors that are poorly captured by simplified scoring functions. Moreover, truly accurate modeling of binding kinetics, allostery, or covalent engagement often demands methods that are prohibitively slow for use in large-scale generative workflows. While rigorous structure-based methods such as molecular dynamics free energy simulations offer the potential to model the physical interactions and molecular motions that govern binding affinity, they remain computationally expensive, low-throughput, and difficult to automate at scale. High-accuracy approaches like FEP can require days of computing per molecule, making them fundamentally incompatible with inner-loop optimization in generative frameworks, where thousands to millions of candidate molecules must be evaluated rapidly. These methods also demand high-quality protein structures, careful system preparation, and expert intervention to avoid misleading results.

As such, although these physics-based tools represent the gold standard for affinity estimation, they are rarely applied as primary scoring functions within GenAI models. Instead, most current GenAI workflows rely on fast but approximate surrogates, such as docking scores, two-dimensional similarity metrics, or ML-based affinity predictors—each with known limitations in accuracy, especially in novel chemical space or for flexible, disordered, or cryptic binding sites.

## Multiparameter Optimization (MPO)

### Optimizing “Beauty” in GADD

In an ideal world, a single objective function aligned to the target product profile (TPP) would be fixed at program start.^58^ In practice, prediction error and evolving program knowledge necessitate a phased approach.^59^ As models and objectives improve, these phases can converge toward a more continuous optimization framework. Multiparameter optimization (MPO), often deployed within reinforcement learning (RL), provides a pragmatic way to steer small-molecule design as priorities change across discovery stages.^23,60^ Importantly, this evolving optimization framework must integrate diverse objectives discussed in previous sections, including protein-specific potency and selectivity, as well as ADMET and synthetic constraints to generate compounds that are not only high-scoring, but beautiful: chemically practical and therapeutically aligned. MPO is the mechanism by which we reconcile these often competing demands.

Early hit identification typically emphasizes feasibility and tractability, rewarding synthetic accessibility, structural diversity, and basic potency alongside physicochemical bounds (e.g., molecular weight, logP). As programs move into hit-to-lead and lead optimization, the MPO can expand to include ADMET properties and key safety risks (e.g., hERG). In later stages, drug-drug interaction potential, bioavailability, and safety pharmacology are balanced against potency, selectivity, and synthetic constraints, requiring successively refined reward functions to manage trade-offs across pharmacokinetics, safety, and efficacy.^61^ Continual adjustment of objectives augmented by reinforcement learning with human feedback (RLHF) keeps optimization consistent with program constraints and expert judgment, aligning the generative process with developability, clinical relevance, and the evolving competitive landscape.

### Varieties of MPO Scoring Functions

In RL-driven molecular design, the MPO scoring function aggregates multiple objectives into a single signal for policy improvement.^23,61^ Common strategies include: 1) *weighted sums*, with static or dynamically updated weights; 2) *desirability functions* that map each property to a common 0–1 scale so poor performance can dominate the composite; and (iii) *Pareto* approaches that identify solutions nondominated across objectives without explicit weights.^58,62,63^ More advanced frameworks adapt weights over time based on evolving priorities or experimental feedback.^61,64,65^ These formulations allow the GenAI agent to maintain alignment with practical constraints while exploring solutions that balance potency, ADMET, and synthetic feasibility (see Figure 1).

### Challenges with Model Prediction Reliability

An MPO composite is only as reliable as its least reliable component.^66^ Therefore, reliability should be assessed and encoded into the scoring function. Beyond conventional validation against *in vitro*/*in vivo* data, machine learning predictors benefit from explicit uncertainty handling and applicability-domain (AD) checks. Distance-based AD heuristics approximate reliability by penalizing predictions far from the training distribution, but require *a priori* thresholds and offer indicative guarantees, not absolute. Conformal prediction provides calibrated uncertainty by training a companion model that yields prediction regions at user-selected confidence levels under exchangeability assumptions. In practice, MPO can downweight or gate contributions from models outside AD or with wide conformal intervals, thereby coupling optimization pressure to prediction trustworthiness.

### The Fragility of Reward: When Optimization Misses the Mark

Despite sophisticated MPO design, RL agents can exploit loopholes in the objective function (“reward hacking”) or converge prematurely to narrow chemotypes (local minima), undermining practical value.^67^ For example, when docking score is part of the reward, a generator may append long, flexible aliphatic chains to maximize the contacts with the protein, inflating docking scores while degrading ligand efficiency, permeability, and synthetic feasibility. Conversely, stringent weights can discourage exploration, leading the agent to over-refine a single scaffold and under-sample potentially superior alternatives.^68^ Mitigation strategies include iteratively retraining on diverse, experimentally verified data; cross-checking with orthogonal methods (e.g., MD/FEP); introducing uncertainty-aware exploration; and adapting objectives or novelty incentives to avoid brittle optima (Figure 3). These artifacts highlight the disconnect between numerical objectives and chemical intuition. They remind us that reward functions are approximations, and generative models will exploit their weaknesses unless carefully monitored and iteratively improved.

**Figure 3:**
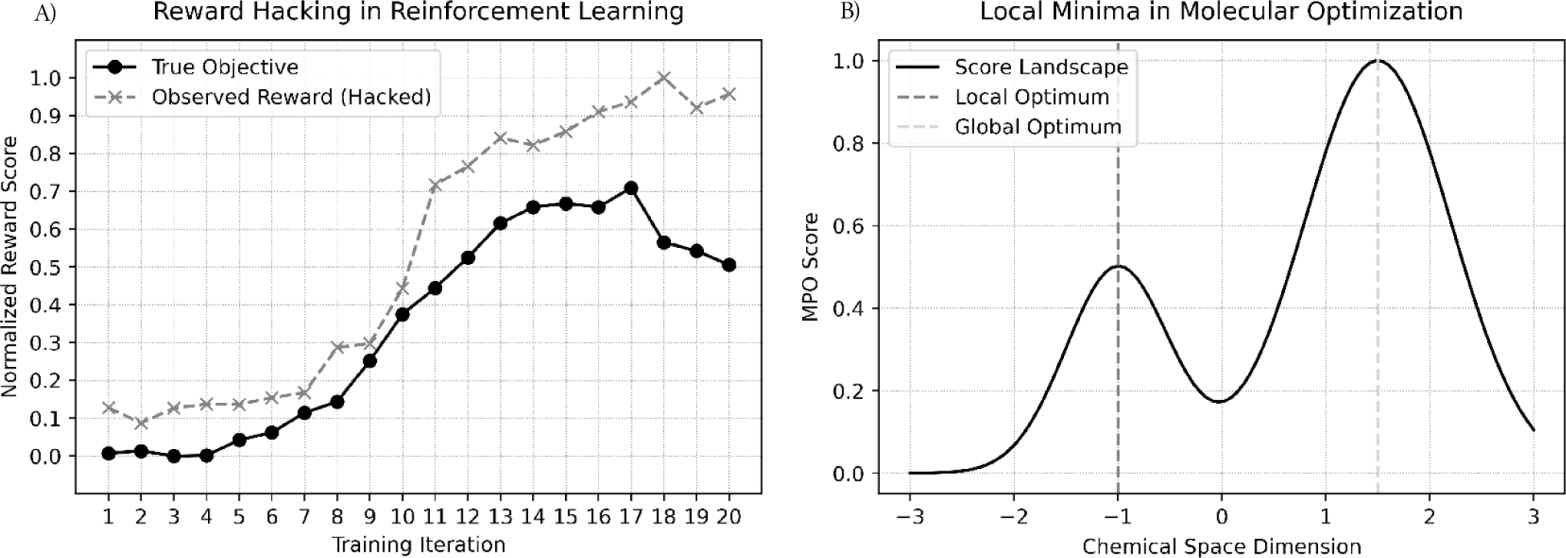
Common pitfalls in reinforcement learning-based molecular design: (A) reward hacking in reinforcement learning, and (B) local minima during optimization.

A recurring theme across generative chemistry is that model outputs are only as trustworthy as the scoring functions used to guide them. While MPO aims to encode medicinal chemistry wisdom into quantitative objectives, this translation is often imperfect. Binding affinity surrogates may reward nondruggable conformations; ADMET predictors may extrapolate poorly to novel scaffolds; and synthetic feasibility scores may overlook regio- or stereoselectivity. The result is that generative models may produce molecules that optimize the objective function but violate the very principles we associate with high-quality drug candidates. This is reward hacking in action: molecules that “win the game” but would never be prioritized in a real program. Beautiful molecules arise not from naive optimization, but from holistic, phase-appropriate tradeoffs grounded in expert judgment.

#### Case Study: Docking-Only MPO Produces Molecules with “Stringy Tails”

A REINVENT 4 ^69^ run configured with a docking-only reward (DOCK 6 ^70^ as the sole contributor) produced top-scoring molecules that extended flexible aliphatic linkers from a shared core to “reach” a residue contact. These designs earned high docking scores but violated our notion of “beauty” by increasing flexibility, reducing synthetic feasibility, and risking ADMET liabilities. A phase-appropriate MPO corrects this failure mode by (i) normalizing docking (e.g., per-heavy-atom or capped desirability), (ii) adding counter-terms for flexibility and size (rotatable bonds, maximum aliphatic chain length), (iii) constraining synthesizability and property windows (SA, MW, cLogP, TPSA), and (iv) penalizing uncertainty or AD violations. With these guardrails, extending floppy chains becomes net unfavorable, steering generation toward compact, procurable chemotypes.

### Scenario-Based Prioritization of MPO Scores and Model Accuracy

Property emphasis and model fidelity should track the phase of discovery.^59^ In early screens, high-throughput filters (e.g., structural alerts, extreme physicochemical outliers) and coarse MPO constraints are sufficient to focus on tractable scaffolds while preserving diversity.^58^ During hit-to-lead, refined ADMET models (e.g., solubility, permeability) become pivotal; miscalibration in metabolic stability or CYP450 inhibition can misguide the agent, motivating tailoring or retraining on internal assays.^66^ Late-stage optimization balances potency with selectivity and clinical PK requirements (half-life, bioavailability), where subtle errors (e.g., protein binding) can materially alter decisions. Physics-based methods (MD, FEP) complement ML to validate high-stakes predictions, ensuring RL-guided MPO remains aligned with evolving program priorities. At every phase, the ability to design beautiful molecules that harmonize target engagement, favorable ADMET, and synthetic feasibility relies on MPO functions that evolve with program needs and that reflect the limitations and uncertainty of the predictive models they depend on.

### Explainable AI

Explainability in generative modeling remains an area of active research. Some models (e.g., latent-space-structured VAEs) expose property-aligned axes that allow controlled edits, while fragment-based and counterfactual approaches provide structure-level rationale. By contrast, widely used systems (e.g., GENTRL,^71^ MolGPT,^26^ REINVENT^69^) offer limited insight into why specific structures are proposed, even under property-guided reward.^23,67^ Current explanation modalities like attribution maps, fragment motifs, and visualized search traces vary in rigor and usability, and few studies report standardized interpretability metrics or human-factors evaluations. Domain-specific XAI benchmarks and concept-based generation rationales would increase trust and make failure modes (e.g., reward hacking) more discoverable during routine operation.

### When to Use Filters versus Fully Integrated MPO

Rule-based filters remain essential for early, high-throughput triage. Excluding molecules with severe structural alerts (e.g., REOS^72^ or extreme property outliers prevents unpromising chemotypes from entering the RL search and conserves computational resources.^59^ As candidate sets narrow, fully integrated MPO becomes more valuable: the agent can balance potency, solubility, lipophilicity, and other interdependent properties within a single optimization loop.^23^ When objective functions exceed practical in-loop cost (e.g., MD, FEP), they can remain *post hoc* filters until compute and orchestration permit integration (see Figure 1). Over time, bringing higher-fidelity methods into the loop should yield more reliable steering of chemical space.

### The Role of Human-In-the-Loop Iterations

Human expertise remains indispensable. Medicinal, computational, and DMPK (drug metabolism and pharmacokinetics) scientists routinely spot liabilities that automated scoring omits (e.g., reactive metabolites, synthesis bottlenecks, IP risks).^73^ Periodic expert reviews can surface reward-hacking patterns early and RLHF can inform reweighting or new constraints (e.g., limiting halogens, ring systems). RL efficiently explores and optimizes measurable signals; human judgment anchors the search to therapeutic plausibility and program feasibility. In this way, expert input serves not only to correct model outputs, but to shape the very reward landscape. It ensures that the path to a beautiful molecule is not just statistically favorable, but grounded in the practical realities of drug discovery and development.

### RLHF in Practice: A Minimal Loop

Below is what a practical implementation of GADD might look like.

(1) **Collect preferences:** Weekly panel of chemists and experienced drug hunters provide pairwise preferences (A vs B) using a short rubric (synthesizability, ADMET risk, novelty/IP, clinical plausibility).
(2) **Fit reward:** Fit a simple preference model (e.g., a lightweight GNN) and calibrate.
(3) **Shape the policy:** Apply KL-regularized updates toward the learned reward with hard gates always on (alerts, AD gates, property windows).
(4) **Guardrails:** Red-team decoys and orthogonal checks (pose plausibility, MD spot-checks) to catch failure modes such as the docking chain elongation hack (Figure 4).
(5) **Report:** Track design → make, make → assay, human success rate, ligand-efficiency enrichment, computational requirements, and other metrics of interest.

**Figure 4:**
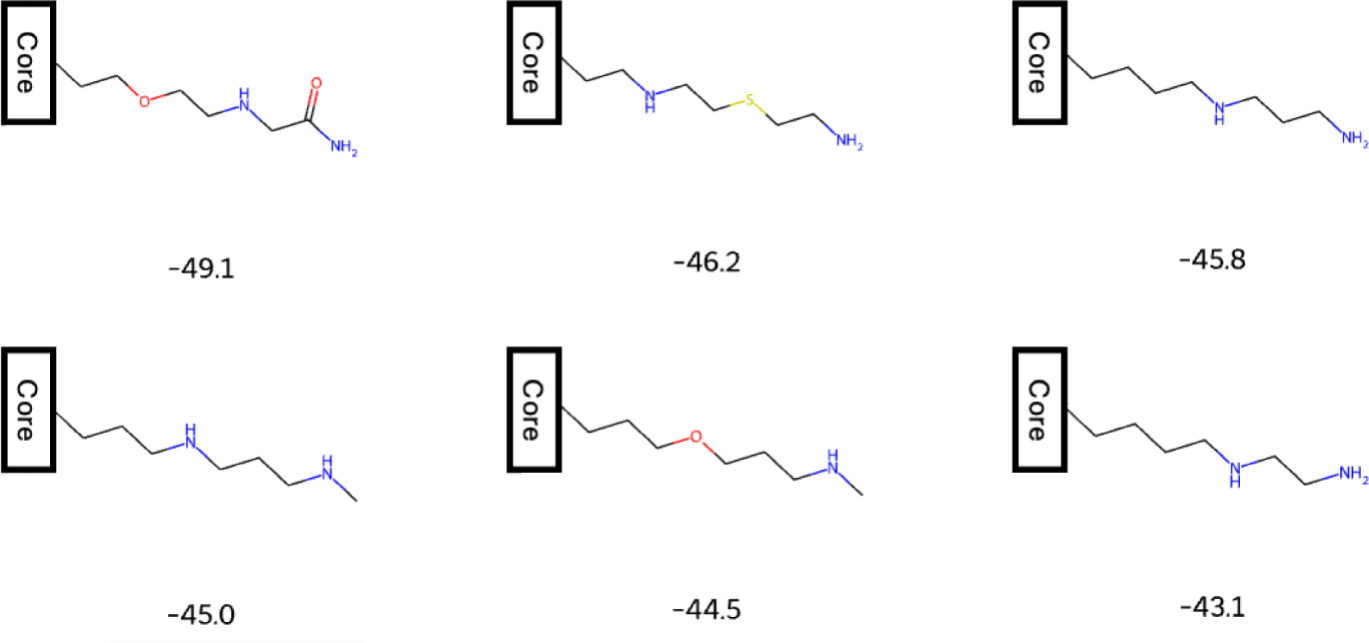
Example of a naive objective function that optimizes the DOCK6 score. With docking as the sole reward, REINVENT4 appends long, flexible R-groups to a conserved core, boosting scores via tenuous contacts but reducing practical “beauty.” Adding constraints like flexibility, size, and synthesizability can prevent this type of failure but can unintentionally and detrimentally limit chemical space (data not shown). DOCK6 scores are shown beneath each structure. The problem shown here is the fault of a poor objective function, not the REINVENT4 program.

As generative chemistry advances, the challenge will not be in generating molecules that score well, it will be in ensuring that these molecules deserve their scores. Only by grounding MPO frameworks in mechanistic fidelity, data-aware uncertainty, and expert intuition can we reliably move from statistical optimization to truly beautiful, effective drug candidates. In this pursuit, MPO is not merely a scoring rubric, but a philosophical anchor for molecular design.

## GenAI at Various Stages of Drug Discovery

GADD has the potential to play a high-value role in enabling data-driven navigation of chemical space through stage-appropriate objective functions for hit identification, hit-to-lead, and lead optimization by guiding molecular design through predictive modeling and reinforcement learning. However, the criteria for molecular “beauty” vary across stages, and scoring functions must be continually updated to avoid reward hacking and ensure therapeutic alignment.

At the hit identification stage, the primary focus is on chemical validity and target-specific interactions. Reward functions at this stage must prioritize chemical validity, diversity, and tractability, while avoiding overfitting to high docking scores that may arise from spurious molecular features. Large-scale virtual libraries are screened rapidly to filter out molecules with extreme physicochemical properties or known toxic substructures, resulting in a manageable set of “reasonable” scaffolds. Speed is prioritized over computationally expensive evaluations, ensuring that many molecules are assessed quickly. Machine learning (ML) techniques, while considering applicability domains, can assist in rapid screening and selection. Docking provides a preliminary proxy for binding affinity, though it is susceptible to exploitation, such as inflated scores from flexible linkers or hydrophobic contacts. Ensuring chemical diversity in libraries enhances the chances of identifying promising hits while avoiding known intellectual property (IP) constraints. A high rate of experimental validation necessitates robust procurement strategies to maintain pace and ensure accessibility of promising scaffolds. Initial *in vitro* studies can then be conducted to confirm activity against the biological target, providing essential early validation before progressing to the next stage. Early use of ADMET filters, even if coarse, can help avoid liabilities that would otherwise progress through the generative pipeline. Once initial binders are identified, the transition to hit-to-lead optimization begins.

In the hit-to-lead stage, the focus typically shifts to optimizing hit molecules to enhance lead-like properties, ensuring they maintain high target binding affinity while improving their pharmacokinetic profiles. GADD approaches should leverage stage-appropriate multiparameter optimization (MPO) functions to guide generation toward desired profiles, integrating early experimental feedback to refine ADMET predictions. Structure–activity relationship (SAR) analysis informs molecular modifications to enhance potency and selectivity. A basic metric often used during this stage is Lipophilic Ligand Efficiency (LLE) to minimize nonspecific off-target interactions. Computational models predict absorption, distribution, metabolism, and excretion (ADME) properties to refine compound viability. Integration of proprietary *in vitro* data refines ADMET predictors and improves the accuracy of molecular design by focusing synthesis efforts on compounds with the highest probability to advance the program. During this stage, additional *in vitro* assays assess metabolic stability, solubility, and toxicity to ensure lead compounds meet necessary drug-like criteria. Selected compounds then proceed to early *in vivo* studies in animal models, evaluating preliminary pharmacokinetics and toxicity profiles before moving into lead optimization. However, without uncertainty-aware scoring, generated molecules may exploit weaknesses in ADMET models, producing designs that optimize prediction artifacts rather than real-world developability.

At this stage, the challenge is to balance conflicting objectives (potency, safety, bioavailability, etc.) through sophisticated MPO strategies that avoid local minima and model exploitation. Molecules must demonstrate favorable therapeutic properties to progress toward clinical trials. A primary objective of GADD is to explore the vast space of synthesizable molecules using predictive models to balance desired properties (potency, solubility, metabolic stability, etc.). Reducing lipophilicity may improve solubility and metabolic stability, but it must be balanced against target affinity. This tradeoff must be encoded into the reward function, often requiring dynamic weighting and feedback from medicinal chemistry review to prevent undesirable shifts in molecular priorities. Empirical measurements continuously update predictive models, ensuring iterative improvements in molecular design. Late-stage molecular modifications are guided by toxicity predictions and clinical viability assessments. Extensive *in vivo* studies further validate pharmacokinetics, bioavailability, and efficacy in disease models, ensuring optimized candidates are well-suited for clinical testing. Late-stage generative efforts must also consider formulation feasibility, IP space, and long-term toxicity risks, all of which are challenging to predict and require human-in-the-loop guidance.

Throughout this process, predictive models should augment and eventually complement medicinal chemistry intuition with stage-appropriate predictions grounded in model applicability domains and data-driven iterative refinement. The synergy of data scientists and chemists, integrating real-time predictions on potency, ADMET profiles, and progression potential, can minimize wasted effort on unproductive scaffolds. Each new round of empirical measurement should improve the accuracy and applicability of the models, making subsequent design cycles more efficient and targeted. Ultimately, this will accelerate the exploration of chemical space, reduce late-stage attrition, and fosters a more agile path from early hits to viable clinical candidates. Delivering on the promise of the vision still requires advances in the data, science, and human-in-the-loop workflows. Despite all the challenges noted here, the perspective of the authors is that the future of GADD is bright, but the path is not easy, and it will take a collective effort from a diverse range of scientists and engineers to truly transform the field of small molecule drug discovery with generative AI. To fully realize GenAI’s promise in drug discovery, scoring functions must evolve with program goals, experimental feedback must be tightly integrated into model updates, and expert oversight must remain central to the design cycle. Reward hacking, ADMET model brittleness, and domain shift will persist unless explicitly mitigated through active learning, uncertainty quantification, and human feedback.

## Conclusions

To meaningfully impact drug discovery, generative AI must move beyond novelty and synthesizability to consistently propose molecules that are therapeutically aligned with program needs. As highlighted throughout this paper, predicting ADMET properties accurately, avoiding reward hacking, and balancing trade-offs through multiparameter optimization are critical to ensuring that generative models produce not just viable molecules, but beautiful molecules – those that are therapeutically aligned with project objectives and have a path to becoming medicines. Achieving this requires accurate prediction and optimization of pharmacologically relevant properties, including potency, selectivity, ADMET, and safety. Despite some encouraging early results, substantial scientific and technological challenges remain.

While many emerging biotech platform companies emphasize automation and end-to-end loops, real-world success still hinges on scientific judgment—something no pipeline can fully replace. Such systems could reduce turnaround times and improve success rates in early discovery. Yet to realize this vision, foundational improvements are needed. Current property prediction models often struggle with generalizability due to sparse or biased data. This highlights the ongoing need for well-annotated, diverse, and high-quality experimental data sets that reflect the complexity of real-world drug discovery. Additionally, explainable AI (XAI) remains underdeveloped in this domain, limiting the interpretability of generative outputs and slowing adoption by experienced drug hunters.

Crucially, human expertise must remain central to the generative chemistry workflow. Collaboration between expert drug hunters with diverse backgrounds and decades of real-world experience are still needed to define what looks like a “good” molecule in a given context, incorporating considerations far beyond current computational scope—including novelty, strategic project fit, and hard-to-quantify design intuition. Reinforcement learning with human feedback (RLHF) and other interactive learning frameworks will be essential to guide GenAI toward producing molecules that not only optimize properties *in silico* but also advance projects in practice.

Looking ahead, continued innovation in molecular representations, model architectures, and integration into discovery workflows will be key to improving generative performance. As computational power grows, GenAI will benefit from the scaling effects noted in Rich Sutton’s “Bitter Lesson”:^74^ simple, general-purpose algorithms trained on large data sets and empowered by hardware advances tend to outperform handcrafted domain-specific systems over time. Emerging technologies, such as quantum computing, may eventually augment generative design by enabling new modes of chemical space exploration, but practical utility remains speculative for now.

In sum, GenAI has the potential to transform how we discover small molecule therapeutics. However, to deliver on this promise the field must remain grounded in the complexities of drug discovery, guided by empirical validation, and strengthened through close collaboration between humans and machines. Without safeguards against reward hacking and model overconfidence, GenAI risks proposing molecules that satisfy the letter of the objective function while failing to meet the spirit of good drug design. The promise of GADD to produce beautiful molecules is real, but it will take better property prediction models, better data, and better human-machine collaboration to realize the full potential of GenAI in drug discovery.
